# Supplementary material for: Small-Molecule Chemical Knockdown of MuRF1 in Melanoma Bearing Mice Attenuates Tumor Cachexia Associated Myopathy
Source: Cells. 2020 Oct 11;9(10):2272. doi: 10.3390/cells9102272 (PMC7600862; doi:10.3390/cells9102272)
Supplement: Supplementary file 1 [file cells-09-02272-s001.zip › Figure S1 - proteomic signature.pptx]

## Slide 1
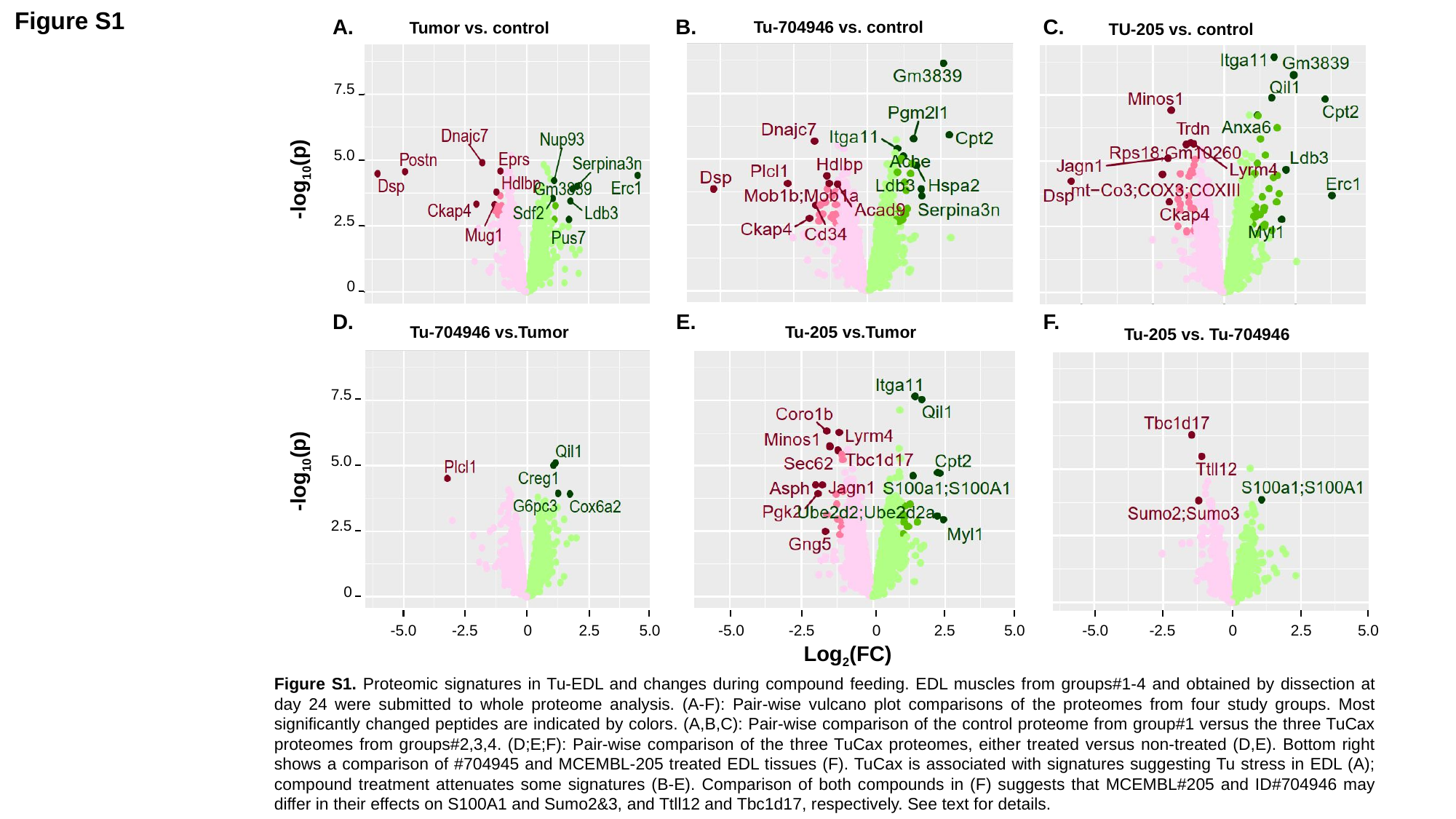

Figure S1
A.
B.
C.
Tu-704946 vs. control
Tumor vs. control
TU-205 vs. control
7.5
5.0
2.5
0
-log10(p)
D.
E.
F.
Tu-704946 vs.Tumor
Tu-205 vs.Tumor
Tu-205 vs. Tu-704946
7.5
5.0
2.5
0
-5.0
-2.5
0
2.5
5.0
-5.0
-2.5
0
2.5
5.0
-5.0
-2.5
0
2.5
5.0
-log10(p)
Log2(FC)
Figure S1. Proteomic signatures in Tu-EDL and changes during compound feeding. EDL muscles from groups#1-4 and obtained by dissection at day 24 were submitted to whole proteome analysis. (A-F): Pair-wise vulcano plot comparisons of the proteomes from four study groups. Most significantly changed peptides are indicated by colors. (A,B,C): Pair-wise comparison of the control proteome from group#1 versus the three TuCax proteomes from groups#2,3,4. (D;E;F): Pair-wise comparison of the three TuCax proteomes, either treated versus non-treated (D,E). Bottom right shows a comparison of #704945 and MCEMBL-205 treated EDL tissues (F). TuCax is associated with signatures suggesting Tu stress in EDL (A); compound treatment attenuates some signatures (B-E). Comparison of both compounds in (F) suggests that MCEMBL#205 and ID#704946 may differ in their effects on S100A1 and Sumo2&3, and Ttll12 and Tbc1d17, respectively. See text for details.
