# Supplementary material for: Small-Molecule Chemical Knockdown of MuRF1 in Melanoma Bearing Mice Attenuates Tumor Cachexia Associated Myopathy
Source: Cells. 2020 Oct 11;9(10):2272. doi: 10.3390/cells9102272 (PMC7600862; doi:10.3390/cells9102272)
Supplement: Supplementary file 1 [file cells-09-02272-s001.zip › Figure S2 - protein expression of selected proteins.pptx]

## Slide 1
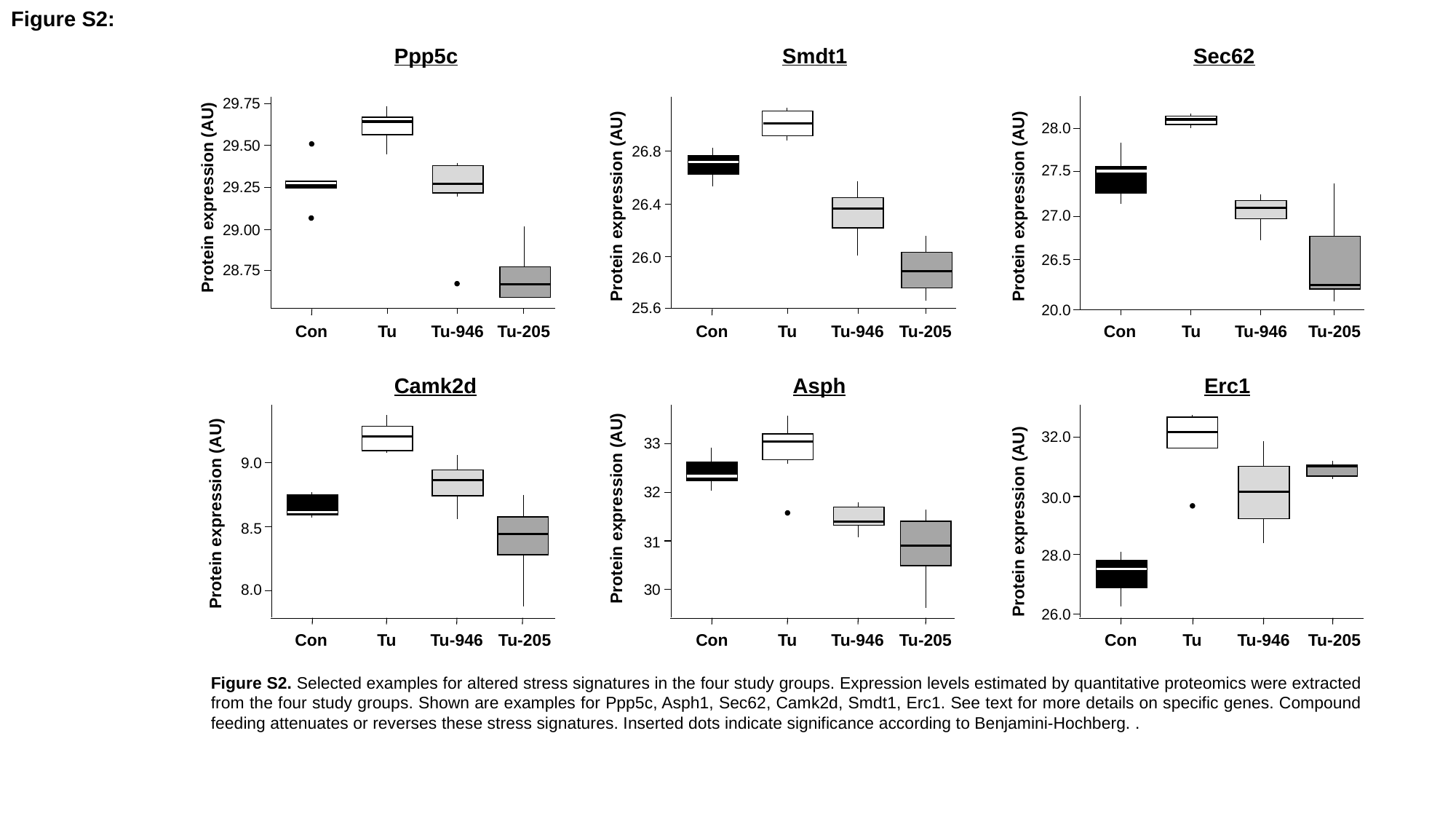

Figure S2:
Ppp5c
29.75
29.50
29.25
Protein expression (AU)
29.00
28.75
Con
Tu
Tu-946
Tu-205
Smdt1
26.8
26.4
Protein expression (AU)
26.0
25.6
Con
Tu
Tu-946
Tu-205
Sec62
28.0
27.5
Protein expression (AU)
27.0
26.5
20.0
Con
Tu
Tu-946
Tu-205
Camk2d
9.0
Protein expression (AU)
8.5
8.0
Con
Tu
Tu-946
Tu-205
Asph
33
32
Protein expression (AU)
31
30
Con
Tu
Tu-946
Tu-205
Erc1
32.0
30.0
Protein expression (AU)
28.0
26.0
Con
Tu
Tu-946
Tu-205
Figure S2. Selected examples for altered stress signatures in the four study groups. Expression levels estimated by quantitative proteomics were extracted from the four study groups. Shown are examples for Ppp5c, Asph1, Sec62, Camk2d, Smdt1, Erc1. See text for more details on specific genes. Compound feeding attenuates or reverses these stress signatures. Inserted dots indicate significance according to Benjamini-Hochberg. .
