# Supplementary material for: Small-Molecule Chemical Knockdown of MuRF1 in Melanoma Bearing Mice Attenuates Tumor Cachexia Associated Myopathy
Source: Cells. 2020 Oct 11;9(10):2272. doi: 10.3390/cells9102272 (PMC7600862; doi:10.3390/cells9102272)
Supplement: Supplementary file 1 [file cells-09-02272-s001.zip › Figure S3 - representative complete western blots.pptx]

## Slide 1
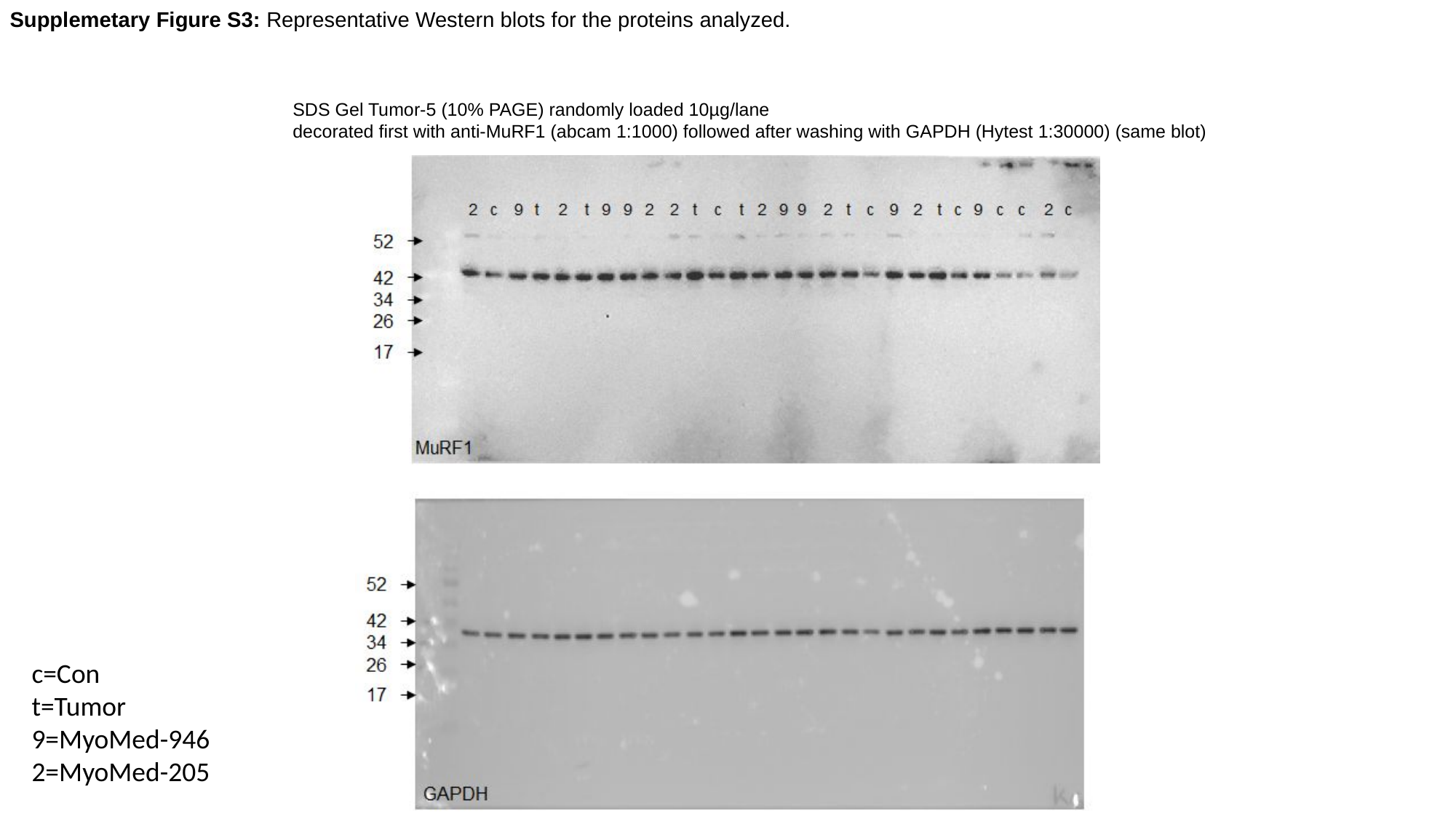

Supplemetary Figure S3: Representative Western blots for the proteins analyzed.
SDS Gel Tumor-5 (10% PAGE) randomly loaded 10µg/lane
decorated first with anti-MuRF1 (abcam 1:1000) followed after washing with GAPDH (Hytest 1:30000) (same blot)
c=Con
t=Tumor
9=MyoMed-946
2=MyoMed-205

## Slide 2
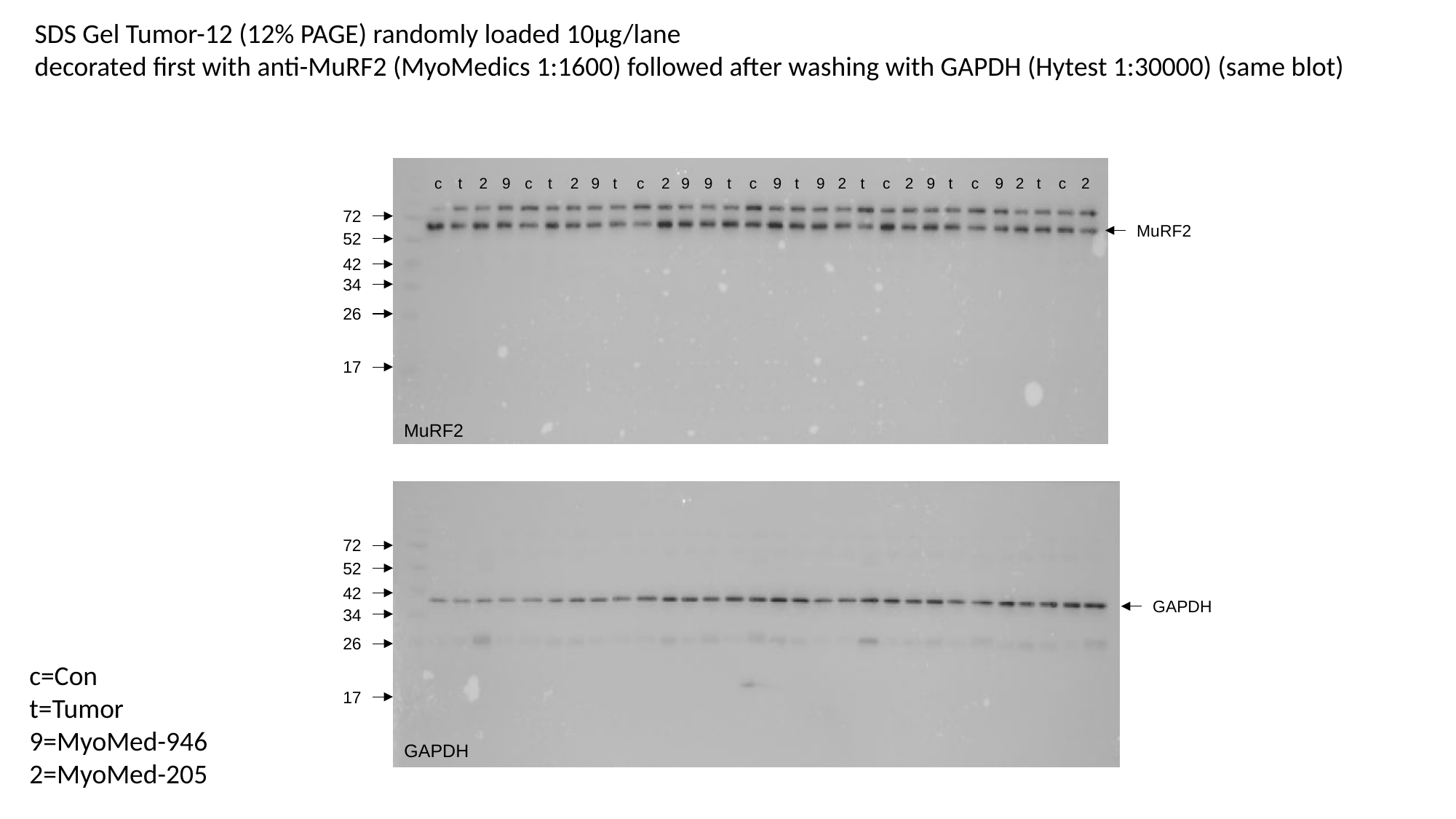

SDS Gel Tumor-12 (12% PAGE) randomly loaded 10µg/lane
decorated first with anti-MuRF2 (MyoMedics 1:1600) followed after washing with GAPDH (Hytest 1:30000) (same blot)
c
t
2
9
c
t
2
9
t
c
2
9
9
t
c
9
t
9
2
t
c
2
9
t
c
9
2
t
c
2
72
MuRF2
52
42
34
26
17
MuRF2
72
52
42
GAPDH
34
26
c=Con
t=Tumor
9=MyoMed-946
2=MyoMed-205
17
GAPDH

## Slide 3
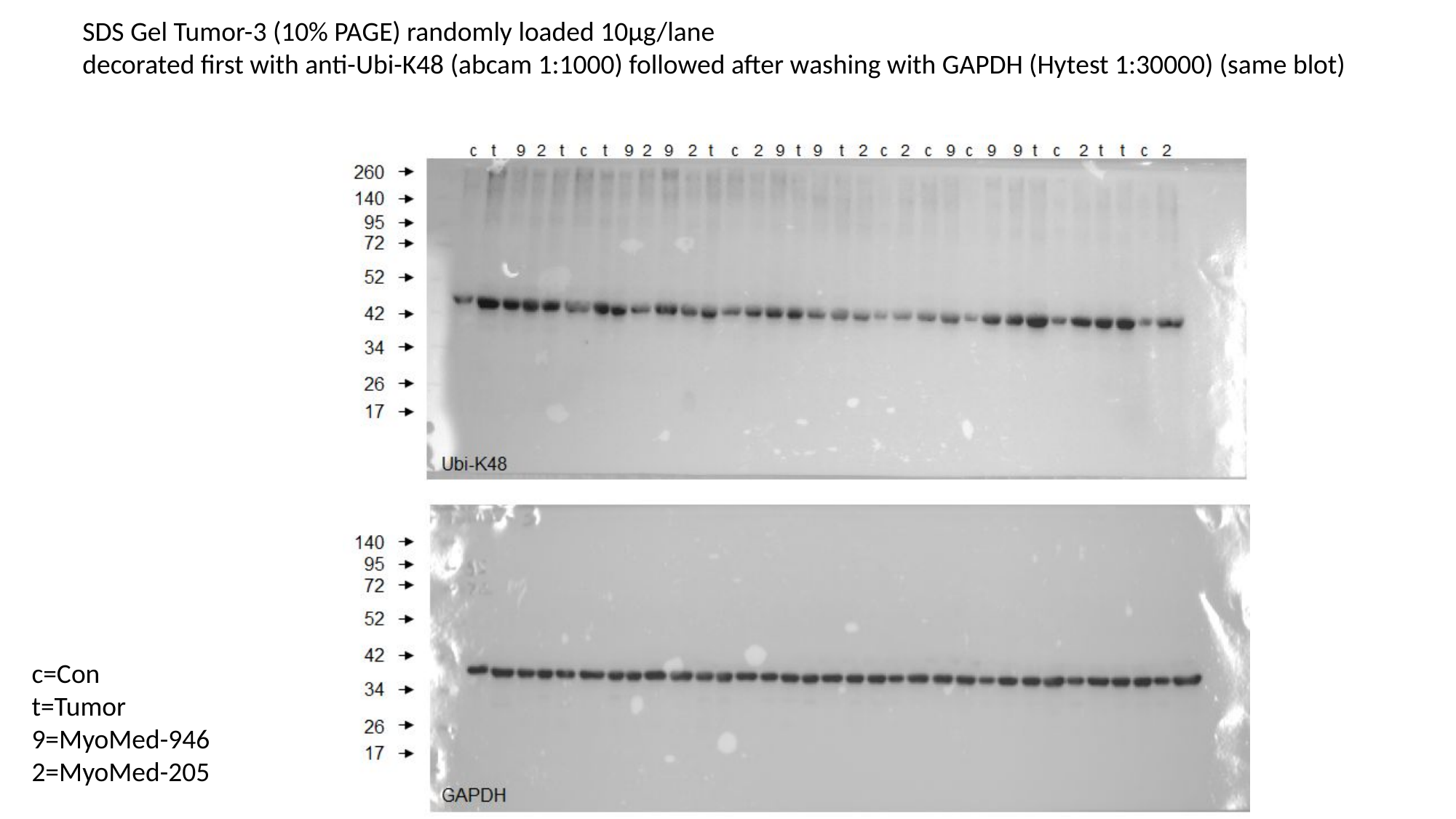

SDS Gel Tumor-3 (10% PAGE) randomly loaded 10µg/lane
decorated first with anti-Ubi-K48 (abcam 1:1000) followed after washing with GAPDH (Hytest 1:30000) (same blot)
c=Con
t=Tumor
9=MyoMed-946
2=MyoMed-205

## Slide 4
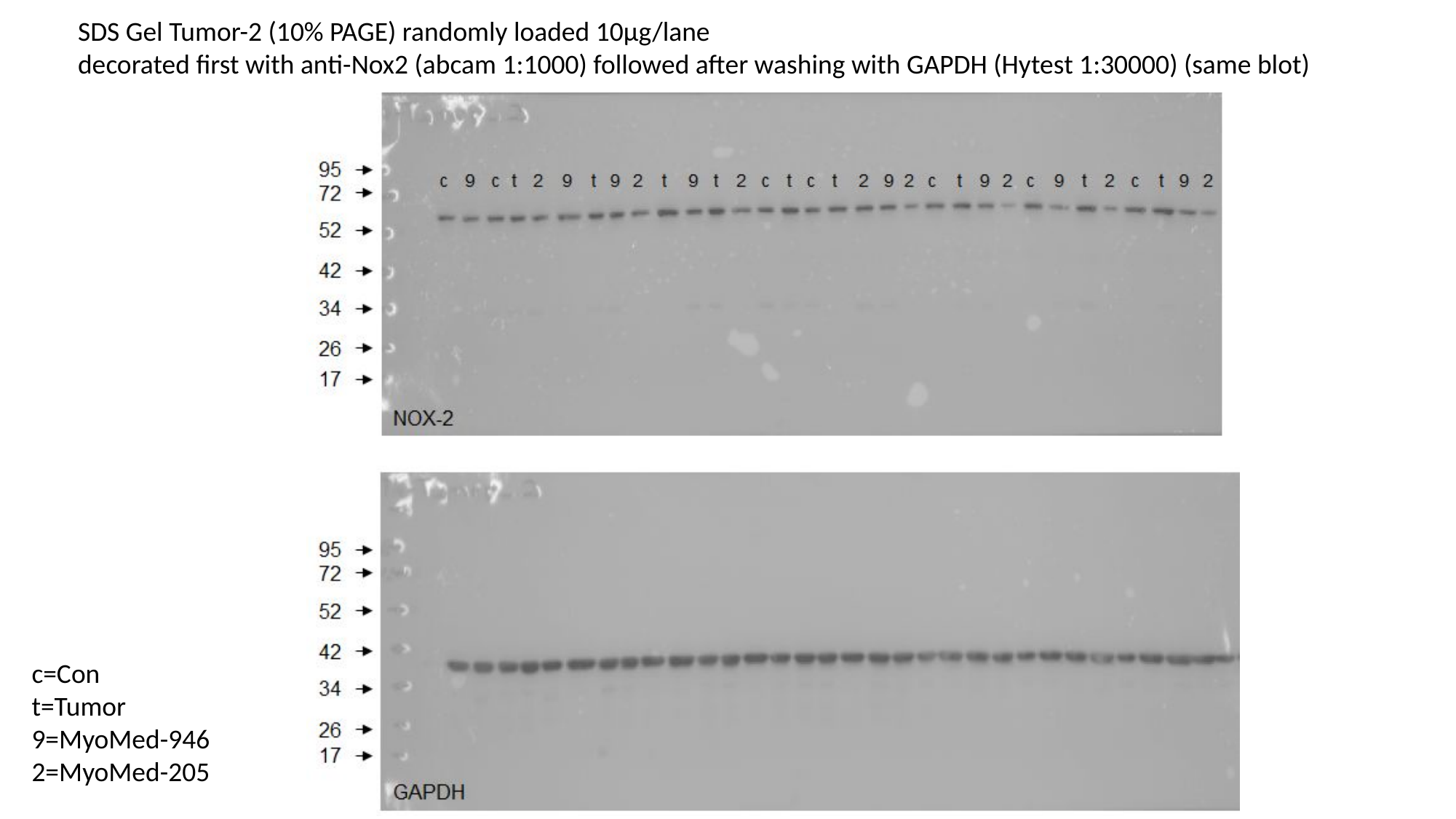

SDS Gel Tumor-2 (10% PAGE) randomly loaded 10µg/lane
decorated first with anti-Nox2 (abcam 1:1000) followed after washing with GAPDH (Hytest 1:30000) (same blot)
c=Con
t=Tumor
9=MyoMed-946
2=MyoMed-205

## Slide 5
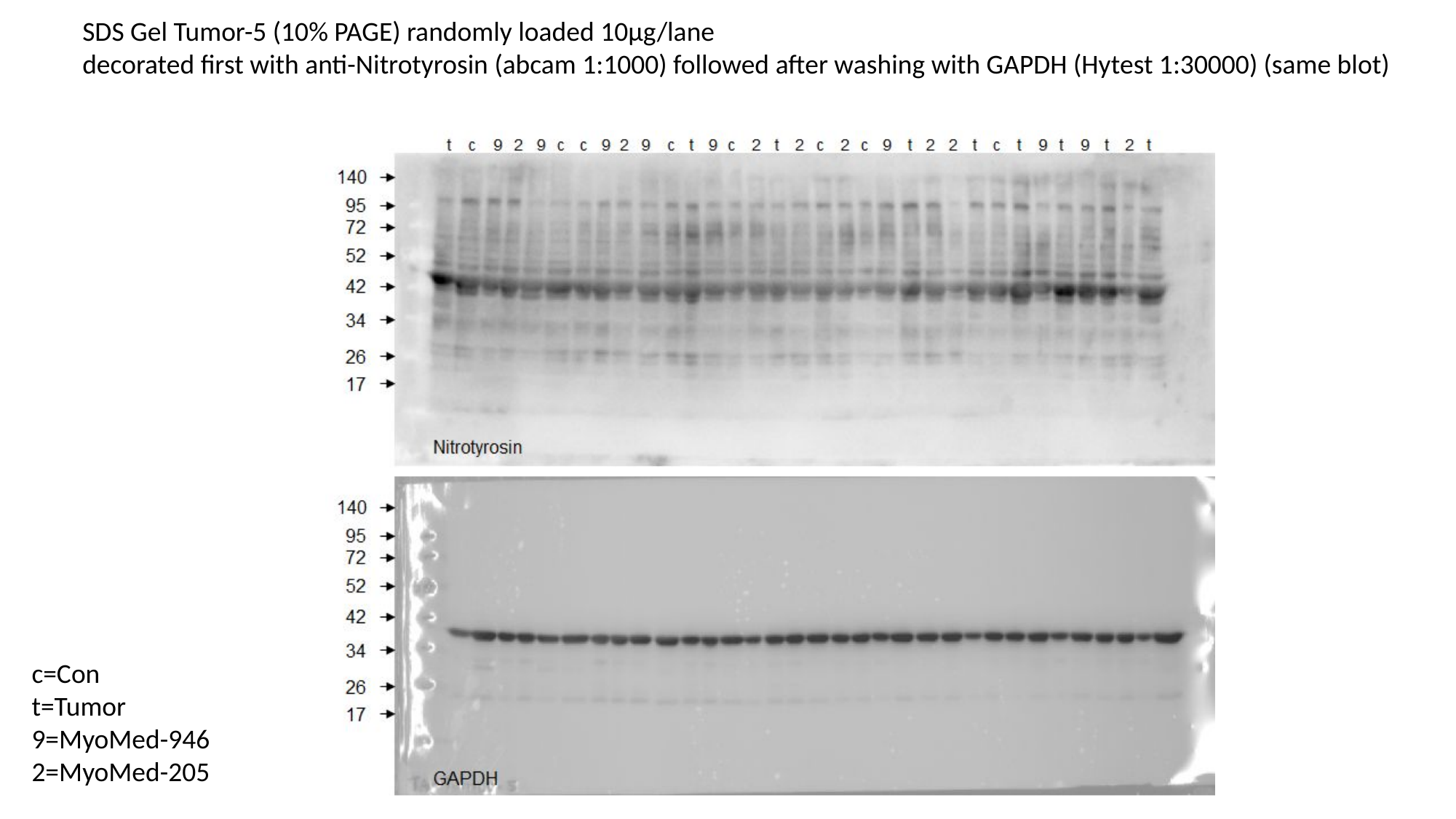

SDS Gel Tumor-5 (10% PAGE) randomly loaded 10µg/lane
decorated first with anti-Nitrotyrosin (abcam 1:1000) followed after washing with GAPDH (Hytest 1:30000) (same blot)
c=Con
t=Tumor
9=MyoMed-946
2=MyoMed-205

## Slide 6
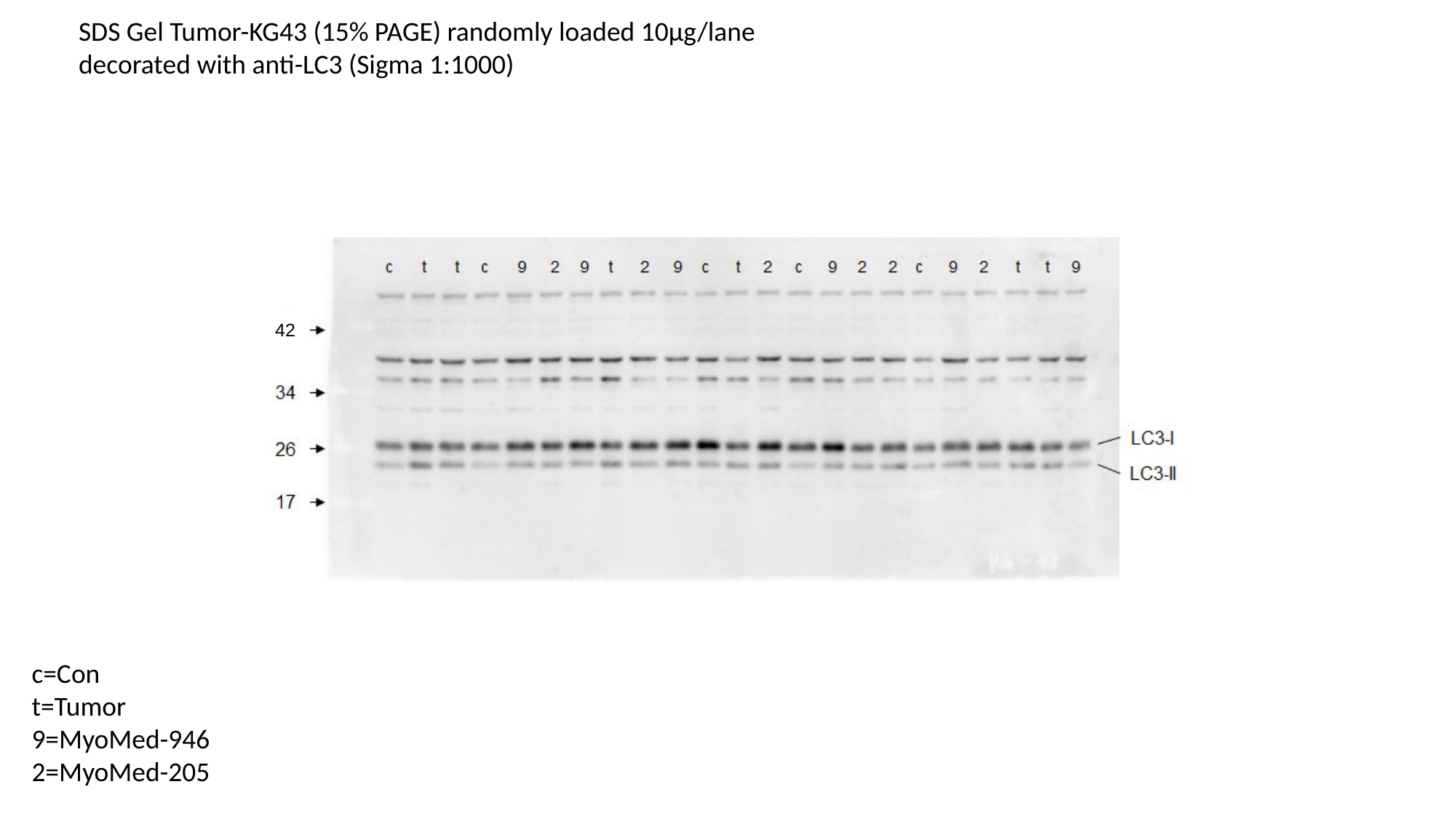

SDS Gel Tumor-KG43 (15% PAGE) randomly loaded 10µg/lane
decorated with anti-LC3 (Sigma 1:1000)
42
c=Con
t=Tumor
9=MyoMed-946
2=MyoMed-205
